# HIV-related stigma trends in the general population of India during an era of antiretroviral treatment expansion, 2005-16

**DOI:** 10.7189/jogh.10.020420

**Published:** 2020-12

**Authors:** Brian T Chan, Venkatesan Chakrapani, Alexander C Tsai

**Affiliations:** 1 Division of Infectious Diseases, Brigham and Women’s Hospital, Boston, Massachusetts, USA; 2 Harvard Medical School, Boston, Massachusetts, USA; 3 Centre for Sexuality and Health Research and Policy (C-SHaRP), Chennai, India; 4 The Humsafar Trust, Mumbai, India; 5 Chester M. Pierce, MD Division of Global Psychiatry, Massachusetts General Hospital, Boston, Massachusetts, USA; 6 Mbarara University of Science and Technology, Mbarara, Uganda

## Abstract

**Background:**

In India, which has the world’s third-largest HIV epidemic, the extent to which levels of HIV-related stigma have changed during an era of ART scale-up is unknown.

**Methods:**

We analyzed data from the 2005-06 and 2015-16 National Family Health Surveys (NFHS) to estimate trends in two stigma domains among people in the general population: desires for social distance from people living with HIV (ie, unwillingness to interact) and fear of serostatus disclosure in the case of a hypothetical HIV infection. We fitted multivariable linear probability models to the data with year of NFHS as the explanatory variable and alternately specifying fear of disclosure or desires for social distance as the dependent variable. Analyses were stratified by sex, state, and high vs low HIV prevalence states.

**Results:**

We included data on 172 795 women and 159 194 men. Desires for social distance declined in 2015-16 compared with 2005-06 (38% in 2015-16 vs 43% in 2005-06; adjusted *b =* -0.046; 95% confidence interval (CI = -0.049 to -0.043; *P* < 0.001) but fear of serostatus disclosure increased (31% in 2005-06 vs 37% in 2015-16; adjusted *b* = 0.058; 95% CI = 0.055-0.062; *P* < 0.001). Declines in social distancing were more pronounced among men and in high HIV prevalence states. Increased fear of serostatus disclosure was greater among women and in high HIV prevalence states. There was significant variability in trends disaggregated by state.

**Conclusions:**

During the first decade of ART scale-up in India, fear of HIV serostatus disclosure in the general population increased despite a decline in desires for social distance.

HIV-related stigma, the social discrediting or devaluation associated with HIV [1], has been associated with reduced uptake of voluntary counseling and testing [2-4], increased high-risk sexual behavior [5,6], reduced likelihood of serostatus disclosure [7-9], and suboptimal uptake of antiretroviral therapy (ART) [10-12]. Consequently, HIV-related stigma has been recognized as a major impediment to HIV prevention and treatment efforts worldwide [13,14] and the UNAIDS 90-90-90 goals [15].

In the general population, dimensions of HIV-related stigma include *negative attitudes* towards people living with HIV (PLHIV)—particularly *desires for social distance* [16] (or unwillingness to interact) which are often related to instrumental fears about casual transmission—that may manifest behaviorally as *enacted stigma* against PLHIV [17]. Additionally, people in the general population may harbor *anticipated stigma*. Although this term is typically applied to persons with a stigmatized attribute [18] who may expect negative consequences such as exclusion or condemnation from others [16], the concept of anticipated stigma can also be applied to members of the general population, who may expect negative consequences should a hypothetical HIV infection be disclosed to others [19-21].

Because so few interventions have been shown to reduce HIV-related stigma on a national or regional scale [22,23], there has been interest on whether ART scale-up itself might reduce levels of HIV-related stigma. On a theoretical level, the impact of widespread access to ART on HIV-related stigma in the general population has been a subject of some debate. On the one hand, it has been argued that ART scale-up might worsen feelings of blaming or moral outrage in the community and create an “ART stigma” [24], wherein the physical health benefits afforded by ART are perceived as allowing PLHIV to renew promiscuous behaviors and transmit HIV to others [25,26]. On the other hand, ART has been argued to reduce stigma through improvements in physical health and HIV-related symptom burden that reduce the extent to which PLHIV internalize stigmatizing beliefs [27,28] and enable the economic rehabilitation and social reintegration of PLHIV [29,30]. This, in combination with the educational campaigns that typically accompany ART scale-up, weakens the symbolic representation of HIV/AIDS as a disease linked with imminent death [31].

Population data from sub-Saharan Africa suggest modest declines in social distancing but an increase in fear of serostatus disclosure during the current era of ART expansion [20,21]. However, although work has been done on HIV-related stigma among PLHIV in India [32] as well as stigma among members of key populations such as men who have sex with men, people who inject drugs, and sex workers [33-35], there are no data on HIV-related stigma trends among the general population in India. This is a significant gap in the literature, given that India has the third-largest HIV epidemic in the world at over 2.1 million PLHIV, highly concentrated among people who inject drugs, transgender women, men who have sex with men, and female sex workers [9,36]. India has significantly expanded access to ART over the last decade in an effort to reach the goal of an AIDS-free generation by 2030 [37], although suboptimal rates of ART uptake and retention in care continue to undermine this goal [9,38,39].

Policymakers need data about the extent to which levels of HIV-related stigma have actually changed in India during the recent era of ART scale-up. If levels of stigma are shown to be decreasing substantially over time, it would suggest that efforts to expand ART access (and concomitant educational and outreach activities) may be effective in reducing stigma. However, if stigma is shown to be stable, declining minimally, or even worsening over time, it would suggest that ART expansion alone is insufficient to mitigate stigma in the community and that further measures are needed to counteract stigma. Disaggregating these trends by state / union territory (hereafter referred to as “state” or “states” for ease of exposition) would highlight the regions where such anti-stigma measures are especially needed. To help fill this gap in knowledge, this study used data from the 2005-06 and 2015-16 India National Family Health Surveys (NFHS) to understand secular trends in HIV-related stigma among the general population in India.

## METHODS

### India National Family Health Surveys

The NFHS is a population-based survey designed to be representative at the national, state, and (for some key indicators) district levels. There have been four iterations of the NFHS, with only the last two surveys (conducted November 2005-August 2006 and January 2015-December 2016) overlapping with ART scale-up. The NFHS use a stratified two-stage cluster sampling design using villages (rural areas) or Census Enumeration Blocks (urban areas) at the first stage followed by a random selection of 22 households at the second stage. By design, the majority of respondents are women. Details of the sampling procedures are included in the full published reports [40]. Ethical approval for each DHS/AIS survey was obtained from appropriate national entities; all data used for this analysis are de-identified and publicly available.

### Measurement of HIV-related stigma

As with other Demographic and Health Surveys [19,20], the standardization of NFHS questions, including those on HIV-related stigma, allows for the analysis of temporal trends in attitudes. The primary outcomes of interest from the NFHS were fear of HIV serostatus disclosure and desires for social distance. Fear of disclosure was assessed by the question “If a member of your family got infected with the AIDS virus, would you want it to remain a secret or not?” Positive responses to this question reflect anticipated stigma in the general population, ie, the expectation of rejection or condemnation if one’s hypothetical stigmatized status were revealed to others [41]. Desires for social distance were assessed by three questions: “If a member of your family became sick with AIDS, would you be willing to care for her or him in your own household?” (a question that may also capture fear of courtesy stigma [1,42]), “Would you buy fresh vegetables from a shopkeeper or vendor if you knew that this person had the AIDS virus?’” and “In your opinion, if a female teacher has the AIDS virus but is not sick, should she be allowed to continue teaching in the school?” Positive responses to these questions reflect desires for social distance from people living with HIV [16], often motivated by instrumental concerns about casual transmission or preoccupations with the symbolic association of HIV with deviance and moral turpitude.

### Statistical analysis

We merged the 2005-06 and 2015-16 NFHS data sets into a single data set, with year of NFHS as a variable. We used two-sample tests of proportion to compare the prevalence of the stigma measures between 2005-06 and 2015-16. To estimate trends using a regression framework, we fitted linear probability models to the data with year of NFHS (2015-16 vs 2005-06) as the primary explanatory variable of interest and alternately specifying fear of disclosure or desires for social distance ( = 1 if participants endorsed any of the three social distance items) as the dependent variable. To account for compositional changes that could potentially confound any observed trends, we adjusted for socio-demographic variables, including age, gender, educational attainment, marital status, household asset wealth [43,44], and employment status. We also adjusted for HIV knowledge, operationalized as the number of correct responses to five questions about HIV prevention and transmission; this variable is identical to the UNAIDS core indicator on comprehensive HIV knowledge [45] (see Appendix S1 in the **Online Supplementary Document**).

We conducted stratified analyses to assess the extent to which stigma trends differed by covariates of potential policy relevance. First, we conducted stratified analyses in which the multivariable regression models were fitted to data from the 29 individual states with data in both the 2005-06 and 2015-16 NFHS. Second, we extracted data on state HIV prevalence and overall country HIV prevalence from the India HIV Estimations 2017 Technical Report [36]. Then we stratified the estimates by low vs high HIV prevalence states, with the cutoff set at the overall HIV prevalence in India in 2017, 0.22%. To formally test the hypothesis that the trends for year of NFHS were different, we fitted a multivariable regression model to the entire sample and included a product term for the interaction between state HIV prevalence (greater than or equal to 0.22% vs less than 0.22%) and year of NFHS.

The alpha level to determine statistical significance was set at the conventional standard of 0.05. Given the large sample sizes, the magnitudes of the estimates are emphasized in the discussion below. All analyses were performed using Stata software (Version 15.0, StataCorp, College Station, TX, USA).

## RESULTS

The overall response rate for both NFHS was greater than 87% for both women and men. We included data from 172 795 women and 159 194 men in the complete-case analyses. Participant characteristics are stratified by gender in **Table 1** and **Table 2**. The three-item social distance scale showed acceptable internal consistency, with a Cronbach’s alpha of 0.70. Overall, women and men appeared to endorse desires for social distance (41% vs 39%; χ^2^ = 250, *P* < 0.001) and fear of serostatus disclosure (35% vs 34%; χ^2^ = 11, *P* = 0.001) to a similar degree.

**Table 1:** Characteristics of female NFHS participants, 2005-06 and 2015-16*

Characteristic	Overall (n = 172 795)	2005-06 (n = 83 826)	2015-16 (n = 88 969)
Age, mean (SD), years	29.2 (9.5)	28.7 (9.3)	29.6 (9.6)
Achieved more than primary education	70%	70%	71%
Married	68%	67%	70%
Household asset index, mean (SD)*	35 126 (92,382)	37 605 (91,297)	32 790 (93,333)
Employed	27%	32%	23%
HIV knowledge score (out of 5), mean (SD)	3.5 (1.4)	3.5 (1.5)	3.5 (1.3)
Endorsed desires for social distance	41%	43%	40%
Endorsed fears of disclosure	35%	31%	38%

*More information about the construction of the household asset index can be found in Filmer & Pritchett (1999,2001). Information about how the household asset index was specifically operationalized in the DHS/AIS is available at: http://www.dhsprogram.com/topics/wealth-index/Index.cfm.,

**Table 2:** Characteristics of male NFHS participants, 2005-06 and 2015-16*

Characteristic	Overall (n = 159 194)	2005-06 (n = 63 445)	2015-16 (n = 95 749)
Age, mean (SD), years	31.2 (10.8)	30.6 (10.6)	31.6 (10.9)
Achieved more than primary education	78%	77%	78%
Married	61%	59%	63%
Household asset index, mean (SD)*	22 693 (93,930)	20 560 (92,262)	24 107 (94,993)
Employed	78%	83%	75%
HIV knowledge score (out of 5), mean (SD)	3.9 (1.2)	3.9 (1.2)	3.8 (1.2)
Endorsed desires for social distance	39%	42%	36%
Endorsed fears of disclosure	34%	32%	35%

SD – standard deviation

*More information about the construction of the household asset index can be found in Filmer & Pritchett (1999,2001). Information about how the household asset index was specifically operationalized in the DHS/AIS is available at: http://www.dhsprogram.com/topics/wealth-index/Index.cfm. All *t* tests / χ-2 tests for differences by gender yielded *P* values of less than 0.001.

The desire for social distance declined from 43% in 2005-06 to 38% in 2015-16 (χ^2^ = 665, *P* < 0.001). The decline was somewhat more pronounced among men (42% to 36%; χ^2^ = 485, *P* < 0.001) than among women (43% to 40%; χ^2^ = 169, *P* < 0.001). Conversely, the fear of serostatus disclosure rose from 31% in 2005-06 to 37% in 2015-16 (χ^2^ = 1100, *P* < 0.001). This rise appeared to be more pronounced among women (31% to 38%; χ^2^ = 1200, *P* < 0.001) than among men (32% to 35%; χ^2^ = 167, *P* < 0.001).

**Figure 1:**
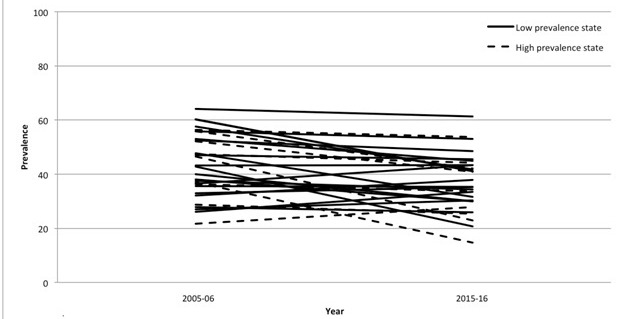
Trends in prevalence of desires for social distance in 29 states / union territories, 2005-2006 to 2015-2016; by state/union territory.

**Figure 2:**
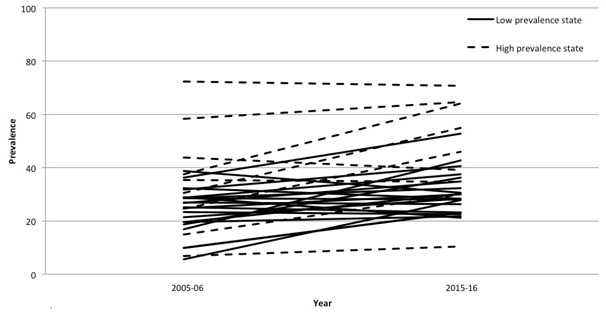
Trends in prevalence of fear of HIV serostatus disclosure in 29 states / union territories, 2005-2006 to 2015-2016; by state/union territory.

Compared with high HIV prevalence states, low HIV prevalence states had less social distancing (39% vs 41%; χ^2^ = 133, *P* < 0.001) and fear of serostatus disclosure (30% vs 43%; χ^2^ = 61,00, *P* < 0.001). Trends in prevalence of stigma by state are shown in **Figure 1** and **Figure 2**, as well as Appendix S1 in the **Online Supplementary Document**. In 2015-16, the percentage endorsing social distancing ranged from 15% (Mizoram) to 61% (Meghalaya) while the percentage endorsing fear of disclosure ranged from 10% (Manipur) to 76% (Puducherry). Of the 29 states with data in both the 2005-06 and 2015-16 NFHS, we found a declining trend in social distancing in 22 (76%) states, with the 2015-16 prevalence of social distancing varying by a factor of four. In contrast, we found an increase in fear of serostatus disclosure in 19 (66%) out of 29 states, with the 2015-16 prevalence of fear of serostatus disclosure varying by a factor of seven.

We estimated a statistically significant negative association between year of NFHS and desires for social distance (adjusted *b =* -0.046; 95% confidence interval (CI) = -0.049 to -0.043; *P* < 0.001) but a statistically significant positive association between year of NFHS and fears of disclosure (adjusted *b* = 0.058; 95% CI = 0.055-0.062; *P* < 0.001) (**Table 3**). These regression coefficients suggest an approximately five percentage-point decrease in desires for social distance and a six percentage-point increase in fear of serostatus disclosure from 2005-06 to 2015-16, after adjustment for covariates, in line with the descriptive statistics described above. These estimates suggest relative changes between 10%-20% compared with the baseline prevalence of the stigma variables.

**Table 3:** Regression estimates for the association between year of National Family Health Survey and stigma variables*

		Unadjusted			Adjusted	
***b***		**95% CI**	***b***	**95% CI**
**Entire country:**						
Expresses desire for social distance from PLHIV	2005-06		Reference		Reference	
	2015-16		-0.044	(-0.048, -0.041)	-0.046	(-0.049, -0.043)
Expresses fear of HIV disclosure	2005-06		Reference		Reference	
	2015-16		0.055	(0.052, 0.059)	0.058	(0.055, 0.062)
**Low prevalence states:**						
Expresses desire for social distance from PLHIV	2005-06		Reference		Reference	
	2015-16		0.88	(0.87, 0.90)	-0.029	(-0.033, -0.025)
Expresses fear of HIV disclosure	2005-06		Reference		Reference	
	2015-16		0.068	(0.064, 0.072)	0.075	(0.071, 0.079)
**High prevalence states:**						
Expresses at least one stigmatizing attitude towards PLHIV	2005-06		Reference		Reference	
	2015-16		-0.063	(-0.069, -0.058)	-0.066	(-0.071, -0.060)
Expresses fear of HIV disclosure	2005-06		Reference		Reference	
	2015-16		0.144	(0.138, 0.150)	0.142	(0.136, 0.148)

CI – confidence interval

*All *P*-values <0.001.

Results for regression models for individual states with data from 2005-06 and 2015-16 are available in Appendix S2 in the **Online Supplementary Document**. There was significant heterogeneity across states, with some states experiencing declines in social distancing and others not, and with some states experiencing increases in disclosure fears and others not.

The magnitude of the negative association between time and desires for social distance was greater in high-prevalence states (adjusted *b =* -0.066; 95% CI = -0.071 to -0.060; *P* < 0.001) compared with low-prevalence states (adjusted *b =* -0.029; 95% CI = -0.033 to -0.025; *P* < 0.001). The magnitude of the positive association between time and fears of disclosure was also greater in high-prevalence states (adjusted *b* = 0.142; 95% CI = 0.136-0.148; *P* < 0.001) compared with low-prevalence states (adjusted *b =* 0.075; 95% CI = 0.071-0.079; *P* < 0.001). These differences in high vs low prevalence states were both statistically significant based on the product term coefficients (*P* < 0.001).

## DISCUSSION

In this analysis of serial cross-sectional data on more than 300 000 persons in the general population of India from 2005-16, a period characterized by significant ART scale-up activity, we found evidence for a decrease in desires for social distance but an increase in fear of HIV serostatus disclosure. These trends were not explained by compositional changes in HIV knowledge or socio-demographic characteristics. More than a third of people in India still express a desire to maintain social distance from PLHIV and anticipate stigma from others in the hypothetical scenario of a family member’s HIV infection. Despite a markedly different socio-cultural environment and an epidemic that is highly concentrated among people who inject drugs, transgender women, men who have sex with men, and female sex workers, our findings are consistent with other studies based on population data from sub-Saharan Africa that have also revealed declining levels of social distancing in the context of increasing disclosure fears [20,21]. These findings suggest that the expansion of ART alone, while important as a structural intervention in and of itself, may be insufficient to normalize HIV and eliminate HIV-related stigma—particularly given the relatively low prevalence of HIV compared to sub-Saharan Africa. Further efforts to counter stigma are likely needed, such as contact interventions [23], changes to laws and policies that maintain structural stigma [10,22], and livelihood interventions that directly address the symbolic implications of living with HIV [31,46].

In addition to the effects of accompanying media and educational campaigns, ART scale-up may reduce desires for social distancing by engendering improvements in physical health and HIV-related symptom burden that enable PLHIV to re-establish themselves as economically productive members of society and reduce the association of HIV with economic incapacity [31,46] and, thereby, the possibility of “social death” [47]. Similar to findings from sub-Saharan Africa [20], we found evidence that there was a more substantial decrease in social distancing in states with a relatively high HIV prevalence compared to states with a relatively low HIV prevalence. One possible reason for this finding is that people in the general population of high prevalence states may have more opportunities to have personal contact with PLHIV who have benefited from ART. Indeed, personal contact with PLHIV has been associated with declines in stigmatizing attitudes in general population samples in sub-Saharan Africa [23]. Another possible reason is that media coverage and educational campaigns may have been more robust in high prevalence states.

It is unclear, however, why fears of HIV serostatus disclosure have increased over time in India generally and why the magnitude of this increase appears to have been greater in states with high prevalence. Because of the HIV knowledge campaigns that have accompanied ART scale-up, people in the general population may feel social pressure to endorse accepting attitudes toward PLHIV without changing their opinions about fear of disclosure of a family member’s hypothetical HIV infection, a scenario that could evoke relatively strong feelings. Theoretically, this effect could have been more pronounced in high-prevalence states where ART scale-up activities may have been more widespread. Alternatively, respondents may have actually changed their attitudes toward PLHIV but perceived that others in the community have not. Such a belief in prevailing injunctive or descriptive norms [48] would necessitate continued vigilance against disclosure of a family member’s hypothetical HIV status for fear of inducing stigma enactments.

Although either of these explanations is plausible, they do not explain why fears of HIV serostatus disclosure appear to have increased. One possible theory is that ART scale-up, while providing health benefits for PLHIV, may not counter and may in fact exacerbate the symbolic association of HIV with deviant behaviors and the sense of blaming or moral outrage in the community. It has been argued that knowledge of ART availability might worsen such moral outrage in that the restoration of physical health from ART might be perceived as allowing PLHIV to engage in promiscuous behaviors and spread HIV to others [25,26]. This theory, which has been suggested by researchers in sub-Saharan Africa, may be equally applicable in India, which has traditionally had a moralistic environment towards behaviors such as sex work and illicit drug use and where adult consensual same-sex relationships have only recently been decriminalized. Finally, it is also possible that trends in disclosure concerns reflect a larger shift in Indian society towards more conservative mores that has occurred over the last decade. The more marked increase in disclosure concerns among women compared to men, for example, could be related to the same societal forces that have contributed to the decline in women’s participation in the labor force over this time period [49].

Our study has several limitations. First, our measures of social distancing and fears of disclosure are self-reports of hypothetical scenarios (rather than validated scales) that could be misinterpreted by participants [50-52]. Furthermore, a single binary measure was used for assessing fears of HIV serostatus disclosure. Indeed, these measures have been previously criticized on these grounds [50,51], and accordingly the DHS (including the NFHS) has revised these stigma indicators to improve their reliability and validity [40]. However, available evidence, albeit from Tanzania, not from India, suggests that these measures have been understood to a reasonable degree by DHS respondents [51]. Additionally, it is important to keep in mind that our analyses emphasize *trends* in these variables; this limitation would only bias our estimates of stigma change if the extent of misinterpretation differed between 2005-06 and 2015-16, which we believe to be unlikely. A second limitation is that the establishment of Telangana in 2014 limits our ability to understand state-level trends in stigma in Andhra Pradesh, as Telangana was formerly the northwestern part of Andhra Pradesh. However, this limitation does not affect our interpretation of country-level trends in stigma or trends in other Indian states.

In conclusion, in this analysis of general population data from the Indian NFHS, we found a modest decline in desires for social distance during a period of significant ART scale-up. Declines in social distancing appeared to be more pronounced in high HIV prevalence states, which may be consistent with the theory that more frequent personal contact with PLHIV who have benefited from the salubrious effects of ART should lessen associations of HIV with inevitable death and assuage fear and misconceptions of PLHIV. Unfortunately, more than a third of Indians still endorse a desire to maintain social distance from PLHIV, and disclosure fears have worsened for reasons that are unclear. Furthermore, declines in social distancing were less pronounced, while increases in fear of serostatus disclosure were more pronounced, among women compared to men. While ART scale-up may be beneficial for at least one form of HIV-related stigma, it is unlikely to be a panacea in terms of eliminating stigma. Further effort is needed to develop and implement effective interventions to counter all forms of HIV-related stigma in all states and among both women and men to help allow the realization of an AIDS-free India.

## Additional material

Online Supplementary Document
