# Supplementary material for: HIV-related stigma trends in the general population of India during an era of antiretroviral treatment expansion, 2005-16
Source: J Glob Health. 2020 Sep 23;10(2):020420. doi: 10.7189/jogh.10.020420 (PMC7698569; doi:10.7189/jogh.10.020420)
Supplement: Online Supplementary Document [file jogh-10-020420-s001.pdf]

Appendix S1: HIV-related stigma by state / union territory, 2005-06 and 2015-16 National Family Health Surveys (NFHS)

States / union territories with HIV prevalence below national prevalence in 2017

State / union territory	Year of NFHS	Percentage endorsing desires for social distance	Percentage endorsing fears of disclosure	HIV prevalence in 2017, percent
Entire country	2005-6	42.5	31.4	0.22
	2015-6	38.1	36.9	
Arunachal Pradesh	2005-6	53.0	18.7	0.06
	2015-6	44.9	36.3	
Assam	2005-6	60.3	9.8	0.06
	2015-6	41.1	23.1	
Bihar	2005-6	32.9	32.3	0.16
	2015-6	35.2	28.3	
Chandigarh	2015-6	15.6	14.3	0.20
Chhattisgarh	2005-6	26.0	19.5	0.13
	2015-6	33.4	30.1	
Dadra and Nagar Haveli	2015-6	44.8	62.5	0.17
Daman and Diu	2015-6	39.2	57.1	0.17
Gujarat	2005-6	47.1	31.7	0.19
	2015-6	45.5	40.5	
Haryana	2005-6	38.0	16.8	0.18
	2015-6	29.9	42.7	
Himachal Pradesh	2005-6	27.0	26.9	0.05
	2015-6	30.3	26.3	
Jammu and Kashmir	2005-6	55.8	26.7	0.03
	2015-6	53.0	34.8	
Jharkhand	2005-6	35.5	24.8	0.14
	2015-6	35.3	29.9	
Kerala	2005-6	47.8	36.2	0.08
	2015-6	31.6	52.7	
Lakshadweep	2015-6	45.7	51.1	n/a
Madhya Pradesh	2005-6	32.1	28.7	0.09
	2015-6	37.8	37.6	
Meghalaya	2005-6	64.1	21.4	0.11
	2015-6	61.3	28.3	
Odisha	2005-6	37.5	5.6	0.13
	2015-6	34.3	27.8	

Puducherry	2015-6	35.8	76.4	0.15
Punjab	2005-6	42.8	25.2	0.18
	2015-6	20.7	23.2	
Rajasthan	2005-6	40.0	38.8	0.10
	2015-6	30.0	30.6	
Sikkim	2005-6	43.2	28.9	0.05
	2015-6	43.4	21.2	
Tripura	2005-6	57.6	19.5	0.09
	2015-6	41.7	21.7	
Uttar Pradesh	2005-6	36.5	28.6	0.09
	2015-6	43.3	28.0	
Uttarakhand	2005-6	27.8	28.5	0.11
	2015-6	25.9	32.3	
West Bengal	2005-6	52.4	23.3	0.2
	2015-6	48.5	22.5	

n/a: not available

States / union territories with HIV prevalence at or above national prevalence in 2017

State / union territory	Year of NFHS	Percentage endorsing desires for social distance	Percentage endorsing fears of disclosure	HIV prevalence in 2017, percent
Entire country	2005-6	42.5	31.4	0.22
	2015-6	38.1	36.9	
Andhra Pradesh	2005-6	55.9	58.3	0.63
	2015-6	42.1	64.7	
Delhi	2005-6	21.6	30.5	0.30
	2015-6	27.9	55.0	
Goa	2005-6	46.5	43.8	0.42
	2015-6	22.9	39.1	
Karnataka	2005-6	52.1	37.5	0.47
	2015-6	40.8	64.1	
Maharashtra	2005-6	36.3	24.6	0.33
	2015-6	33.7	46.0	
Manipur	2005-6	28.8	6.8	1.43
	2015-6	25.3	10.4	
Mizoram	2005-6	37.0	35.3	2.04
	2015-6	14.7	34.7	
Nagaland	2005-6	56.4	14.9	1.15
	2015-6	53.7	29.9	
Tamil Nadu	2005-6	47.4	72.3	0.22

	2015-6	44.2	70.7	
Telangana	2015-6	51.9	66.0	0.70

Appendix S2: Regression estimates for the association between year of National Family Health Survey and stigma variables, by state / union territory (for states / union territories with data in 2005-06 and 2015-16)

States / union territories with HIV prevalence below national prevalence in 2017

State / union territory	Adjusted *b*, desires for social distance	95% Confidence Interval	Adjusted *b*, fear of serostatus disclosure	95% Confidence Interval
Entire country	*-0.046*	*-0.049 to -0.043*	*0.058*	*0.055 to 0.062*
Arunachal Pradesh	-0.025	-0.056 to 0.005	0.175	0.147 to 0.203
Assam	-0.180	-0.200 to -0.161	0.137	0.123 to 0.152
Bihar	-0.003	-0.023 to 0.017	-0.016	-0.038 to 0.005
Chhattisgarh	0.051	0.032 to 0.070	0.110	0.091 to 0.129
Gujarat	-0.015	-0.035 to 0.005	0.105	0.085 to 0.125
Haryana	-0.047	-0.068 to -0.025	0.255	0.234 to 0.276
Himachal Pradesh	0.021	0.002 to 0.039	-0.0005	-0.020 to 0.019
Jammu and Kashmir	0.092	0.074 to 0.110	-0.015	-0.033 to 0.003
Jharkhand	-0.078	-0.102 to -0.054	0.052	0.028 to 0.075
Kerala	-0.114	-0.136 to -0.093	0.143	0.120 to 0.166
Madhya Pradesh	0.003	-0.010 to 0.017	0.078	0.064 to 0.093
Meghalaya	-0.035	-0.068 to -0.002	0.088	0.058 to 0.117
Odisha	-0.007	-0.024 to 0.009	0.230	0.218 to 0.242
Punjab	-0.128	-0.147 to -0.108	0.007	-0.012 to 0.026
Rajasthan	-0.115	-0.134 to -0.095	-0.074	-0.095 to -0.053
Sikkim	-0.009	-0.040 to 0.023	-0.050	-0.078 to -0.021
Tripura	-0.060	-0.090 to -0.029	0.025	-0.002 to 0.052
Uttar Pradesh	0.056	0.046 to 0.065	-0.011	-0.020 to -0.001
Uttarakhand	-0.019	-0.040 to 0.002	0.049	0.025 to 0.072
West Bengal	-0.048	-0.067 to -0.029	0.006	-0.011 to 0.023

States / union territories with HIV prevalence at or above national prevalence in 2017

State / union territory	Adjusted *b*, desires for social distance	95% Confidence Interval	Adjusted *b*, fear of serostatus disclosure	95% Confidence Interval
Entire country	*-0.046*	*-0.049 to -0.043*	*0.058*	*0.055 to 0.062*
Andhra Pradesh	-0.094	-0.113 to -0.076	0.059	0.039 to 0.078
Delhi	0.028	0.002 to 0.054	0.230	0.199 to 0.261
Goa	-0.192	-0.219 to -0.165	-0.049	-0.080 to -0.019
Karnataka	-0.112	-0.127 to -0.097	0.272	0.256 to 0.287

Maharashtra	-0.044	-0.056 to -0.032	0.217	0.205 to 0.230
Manipur	-0.046	-0.061 to -0.030	0.036	0.025 to 0.047
Mizoram	-0.226	-0.251 to -0.202	0.006	-0.022 to 0.034
Nagaland	-0.071	-0.093 to -0.050	0.149	0.129 to 0.169
Tamil Nadu	-0.008	-0.021 to 0.005	-0.021	-0.033 to -0.008
